# *Rubus occidentalis* alleviates hyperalgesia induced by repeated intramuscular injection of acidic saline in rats

**DOI:** 10.1186/s12906-016-1192-z

**Published:** 2016-07-11

**Authors:** Geun Joo Choi, Hyun Kang, Won Joong Kim, Chong Wha Baek, Yong Hun Jung, Young Cheol Woo, Ji Wung Kwon

**Affiliations:** Department of Anesthesiology and Pain Medicine, College of Medicine, Chung-Ang University, 84 Heukseok-ro, Dongjak-gu, Seoul, 06911 Republic of Korea; Gochang Berry & Bio Food Research Institute, Gochang-gun, Jeollabuk-do Korea; Department of Anesthesiology and Pain Medicine, Ewha Womans University School of Medicine, Seoul, Korea

**Keywords:** *Rubus occidentalis*, Black raspberry, Chronic pain, Antinociception

## Abstract

**Background:**

The purpose of this study was to evaluate the antinociceptive effect of black raspberry (*Rubus occidentalis*) fruit extract (ROE) in a rat model of chronic muscle pain and examine the mechanisms involved.

**Methods:**

Adult male Sprague–Dawley rats were used, and chronic muscle pain was induced by two injections of acidic saline into one gastrocnemius muscle. For the first experiment, 50 rats were randomly assigned to five groups. After the development of hyperalgesia, rats were injected intraperitoneally with 0.9 % saline or ROE (10, 30, 100, or 300 mg/kg). For the second experiment, 70 rats were randomly assigned to seven groups. Rats were injected intraperitoneally with saline, yohimbine, dexmedetomidine, prazosin, atropine, mecamylamine, or naloxone after the development of hyperalgesia. Ten minutes later, ROE (300 mg/kg) was administered intraperitoneally. For both experiments, the mechanical withdrawal threshold (MWT) was evaluated with von Frey filaments before the first acidic saline injection, 24 h after the second injection, and at 15, 30, 45, 60, 80, 100, and 120 min, 24 and 48 h after the drug administration.

**Results:**

Compared with the control group, the MWT significantly increased up to 45 min after injection of ROE 100 mg/kg and up to 60 min after injection of ROE 300 mg/kg, respectively. Injection of ROE together with yohimbine or mecamylamine significantly decreased the MWT compared with the effect of ROE alone, while ROE together with dexmedetomidine significantly increased the MWT.

**Conclusions:**

ROE showed antinociceptive activity against induced chronic muscle pain, which may be mediated by α2-adrenergic and nicotinic cholinergic receptors.

**Electronic supplementary material:**

The online version of this article (doi:10.1186/s12906-016-1192-z) contains supplementary material, which is available to authorized users.

## Background

Musculoskeletal pain is a significant health issue with considerable costs and impact for individuals and society. Chronic pain and hyperalgesia induced by musculoskeletal injuries are managed using a multidisciplinary approach, but can be disabling and difficult to treat [1]. Recently, food-based approaches for chronic pain management have been investigated. Among them, blackberry fruits (*Rubus* spp.), has increasingly drawn attention owing to their high content of anthocyanins and other phenolic compounds [2]. Anthocyanins, a class of flavonoids, have anti-inflammatory, antioxidant, antimicrobial, and anti-carcinogenic properties [3–5], which suggest that *Rubus* fruits have a high potential for medical application.

*Rubus occidentalis*, commonly called black raspberry, has one of the highest anthocyanin contents among *Rubus* spp. and a potent antioxidant capacity [6, 7]. Oxidative stress could play a role in chronic pain, especially in the musculoskeletal system [8], and the strong antioxidant action of *R. occidentalis* indicates its therapeutic potential. In addition, studies have reported antinociceptive effects of other *Rubus* species [9, 10].

We hypothesized that *R. occidentalis* extract (ROE) would be beneficial for chronic musculoskeletal pain. To test this hypothesis, we used a rat model of long-lasting hyperalgesia induced by two intramuscular injections of acidic saline, which produces bilateral muscle and cutaneous mechanical hypersensitivity [11, 12].

The primary outcome of this study was to evaluate the antinociceptive effect of *R. occidentalis* in chronic muscle pain. The secondary outcome was to assess the mechanisms involved in its analgesic activity.

## Methods

The present study was performed and described according to the Animal Research: Reporting In Vivo Experiments (ARRIVE) statement [13].

### Preparation of ROE

The ROE was provided by Gochang Berry & Bio Food Research Institute (Gochang-gun, Jeollabuk-do, Korea). Mature fruits of *R. occidentalis* were collected from Gochang-gun in South Korea and stored at −20 °C. In brief, the fruits were extracted twice with 50 % ethanol at 80 °C by using a reflux condenser, and extracts were filtered and concentrated. The concentrate was lyophilized in a freeze-dryer and stored at −20 °C until use. All procedures were conducted under sterilized condition, and stored ROE was used just before intraperitoneal injection.

### Study animals

The experiment was approved by the Institutional Animal Care and Use Committee of Chung-Ang University (No. 2015–00009). All experiments were performed in accordance with the National Institutes of Health Guide for the Care and Use of Laboratory Animals. Adult male Sprague–Dawley rats (250–300 g; Coretec, Seoul, Korea) were single-housed in cages in a temperature-controlled room (22 °C) and fed a standard laboratory diet and tap water. They were kept under a 12 h light/dark cycle (lights on from 8:00 a.m. to 8:00 p.m.) and acclimated to the housing facilities for 1 week prior to commencing experimental procedures. Rats showing any abnormalities were excluded.

### Induction of hyperalgesia in muscle

All procedures were performed under sterile conditions by an investigator who was unaware of the group allocations of the individual rats. Immediately after baseline behavioral measurements as described below, rats were anesthetized briefly with 1–4 % isoflurane in 100 % oxygen and injected with 100 μL of pH 4.0 preservative-free sterile saline into one lateral gastrocnemius muscle on day 0 and again on day 3. The pH was adjusted to within 3.9 to 4.1 with hydrochloric acid using a SevenEasy Mettler Toledo pH meter (Mettler Toledo International, Greifensee, Switzerland) [11].

### Drug preparation and administration

One researcher, who was not involved in this study, prepared syringes containing various doses of ROE which were dissolved in 2 mL of normal saline for ROE groups. Syringes containing 2 mL of normal saline were prepared for the control group. The syringes were covered with opaque tape and numbered sequentially according to a randomization list generated using PASS™ 11 software (NCSS, Kaysville, UT, USA). As intraperitoneal injection is the most frequently used parenteral route of administration in rats, drugs were intraperitoneally administered according to the study protocol. All experimental procedures were conducted by researchers who were blinded to the group allocation of each animal.

### Experiment 1: Evaluation of antinociceptive effect of ROE

The purpose of experiment 1 was to evaluate the anti-nociceptive effect of ROE on induced chronic muscle pain. Fifty rats were randomly assigned to one of five groups of ten rats (control and 10, 30, 100, and 300 mg/kg ROE). The dose level of ROE was referred to the amounts used in other experimental studies on the pharmacological effect of *Rubus* spp. [14, 15], and fixed based on logarithmic increase. Various doses of ROE or normal saline were injected intraperitoneally 24 h after the second injection of acidic saline.

In addition, ketorolac of 10 mg/kg was injected intraperitoneally in ten rats for the positive control [16]. We used ten naive rats with no injection of acidic saline for the negative control.

### Experiment 2: Elucidation of mechanism mediating ROE-induced antinociception

The purpose of experiment 2 was to examine whether the effects of ROE on mechanical hyperalgesia induced by repeated intramuscular injections of acidic saline were mediated by α (1 and 2)-adrenergic, cholinergic (nicotinic and muscarinic), and/or opioid receptors. The use of drugs administered to elucidate the possible involvement of those receptor systems was based on previous studies [17–20]. The study drugs were purchased from Sigma Aldrich (U.S.A.). Seventy rats were randomly assigned to one of seven groups of ten rats, which were injected with either ROE only (control group) or ROE with normal saline, yohimbine 2 mg/kg, dexmedetomidine 50 μg/kg, prazosin 1 mg/kg, atropine 5 mg/kg, mecamylamine 1 mg/kg, or naloxone 5 mg/kg, 24 h after the development of hyperalgesia. Ten minutes later, 300 mg/kg of ROE was injected intraperitoneally.

### Behavioral measurements

For experiments 1 and 2, individual rats were placed on an elevated plastic mesh floor (8 × 8 mm perforations) under an overturned clear plastic cage (21 × 27 × 15 cm) and allowed to acclimate for 15 min. The rats were then tested to determine their withdrawal thresholds to mechanical stimulation using von Frey filaments (Stoelting Co., Wood Dale, IL, USA). The filaments were applied vertically to the plantar aspect of the hindpaw by administering pressure sufficient to gently bend the filament. Filaments with bending forces of 4, 9, 20, 59, 78, 98, 147, and 254 mN were progressively applied until the hindpaw was withdrawn or a bending force of 254 mN (the cutoff value) was reached. Each filament was applied three times at intervals of 3 min. The lowest bending force that caused paw withdrawal was recorded as the mechanical withdrawal threshold (MWT). After a response was observed, filaments with higher and lower bending forces were tested to confirm the MWT. In all groups, the MWT was assessed before first injection (BI), 24 h after second injection (AI), and 15, 30, 45, 60, 80, 100, and 120 min, 24 and 48 h after the injection of the test drugs.

### Motor function tests

In order to identify the effect of ROE on motor function or the sedative effect of ROE, we used an accelerating Rota-Rod treadmill (Jeung-do Bio & Plant Co., Ltd., Seoul, Korea). Fifteen rats were randomly assigned to one of five groups of three rats (control and ROE 10, 30, 100, and 300 mg/kg). In addition, six rats were used in Naive and Ketorolac 10 mg/kg groups as positive and negative controls, respectively. The Rota-Rod test was performed 30 min after the intraperitoneal administration of ROE. The rats were placed on the Rota-Rod treadmill and its speed was gradually increased from one to 18 rotations per minute (rpm) for 120 s and maintained for another 30 s at 18 rpm [11]. The time at which the rat fell off the Rota-Rod was noted.

### Sample size calculation

The primary outcome measure of this study was MWT as assessed using von Frey filaments. To estimate the required group size for experiment 1, a pilot study was conducted to measure MWT in 6 hyperalgesia-induced rats (control group). The averages of the MWT at BI, AI, 15, 30, 45, 60, 80, 100, and 120 min, and 24 and 48 h after injection of normal saline were 91.5, 20.1, 20.1, 27.4, 27.4, 27.4, 33.0, 27.4, 33.0, 33.0, and 33.0 mN, respectively. The standard deviations of MWT ranged from 5.9–20.6 mN, and autocorrelation between adjacent measurements on the same subject was approximately 0.7. For our power calculation, we assumed that first-order autocorrelation adequately represented the autocorrelation pattern. We wanted to detect 10, 20, 30, and 40 % increases in MWT in the ROE 10, 30, 100, and 300 mg/kg groups, respectively, compared with that in the control group. With an α of 0.05 and a power of 80 %, we calculated that we needed nine rats per group. Allowing for a drop-out rate of 10 %, we included ten rats per group.

For experiment 2, sample size estimation was performed using the results of the study assessing the antinociceptive effect of ROE. The average MWT in the 300 mg/kg ROE group at BI, AI, 15, 30, 45, 60, 80, 100, and 120 min, and 24 and 48 h after injection of normal saline were 94.1, 28.8, 59.4, 64.2, 63.9, 53.3, 50.0, 43.3, 39.4, 35.5, 32.1, and 45.5 mN, respectively. The standard deviations of MWT ranged from 10.5–30.6 mN, and autocorrelation between adjacent measurements on the same subject was approximately 0.7. For our power calculation, we assumed that first-order autocorrelation adequately represented the autocorrelation pattern. We aimed to detect a 20 % decrease of MWT in the ROE with yohimbine, mecamylamine, and dexmedetomidine groups and a 10 % decrease in the ROE with naloxone, atropine, and prazosin groups. With an α of 0.05 and a power of 80 %, we calculated that we needed eight rats per group. Allowing for a drop-out rate of 10 %, we included ten rats per group. PASS 11™ software (NCSS) was used to calculate the sample size.

### Statistical analysis

The Shapiro-Wilk test and q-q plot was used to test the normality of variables. MWT were analyzed using repeated measures of ANOVA followed by Tukey test, and Rota-Rod test were compared using the Kruskal-Wallis test followed by Bonferroni correction. Individual measurements were expressed as mean ± standard error and were analyzed using SPSS 20.0 (IBM, Armonk, NY, USA). A p value of less than 0.05 was considered statistically significant.

## Results

**Fig. 1:**
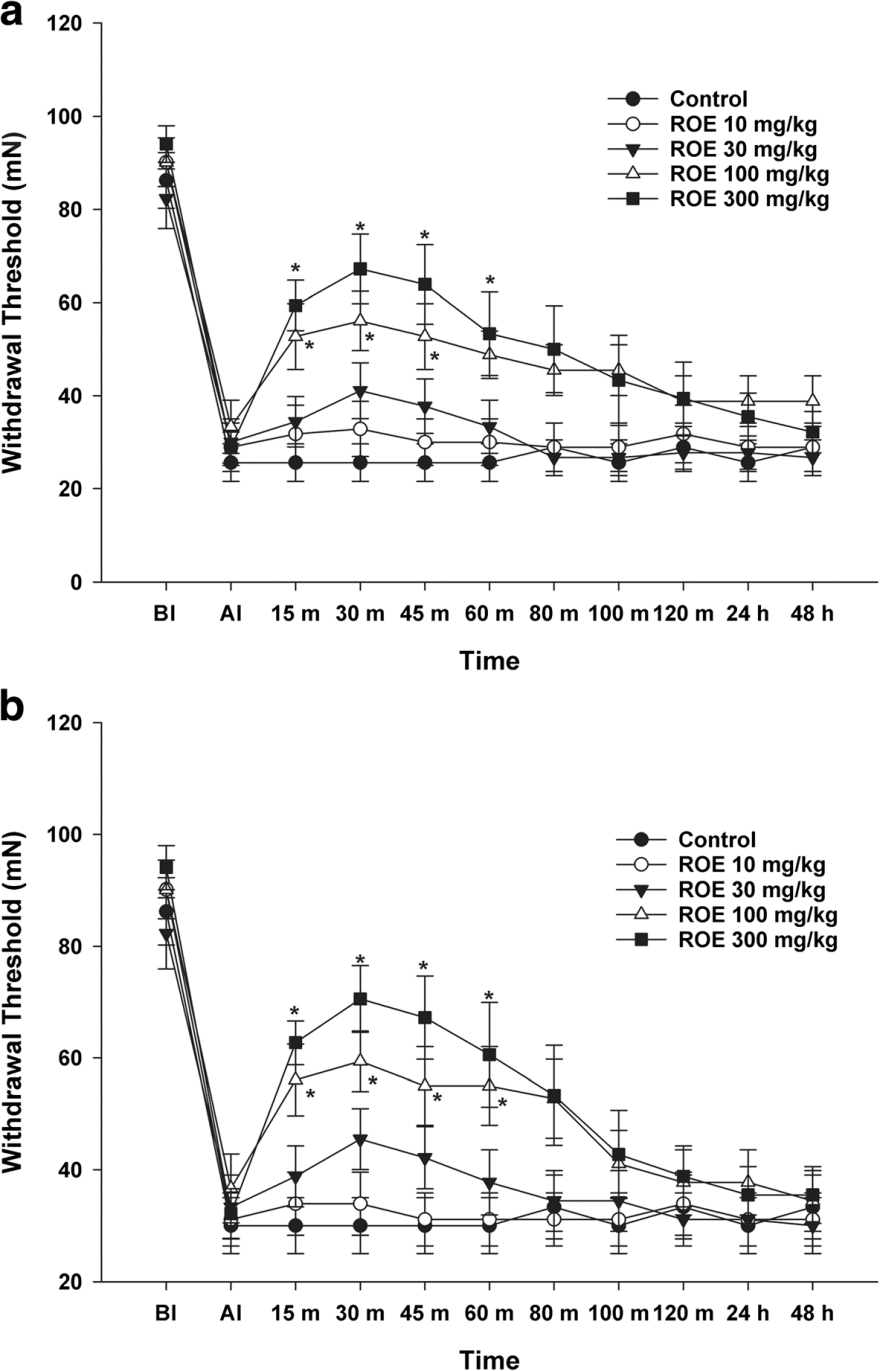
Antinociceptive effect of *Rubus occidentalis* extract (**a**. ipsilateral site, **b**. contralateral site). Rats were injected intraperitoneally with ROE at the indicated doses after the development of hyperalgesia. ROE, *Rubus occidentalis* extract; BI, before injection; AI, after injection, *p < 0.05 compared with the control group. Data were analyzed using repeated measures of ANOVA followed by Tukey test

Throughout the experimental period, all rats remained well-groomed and seemed to maintain normal food and water intake, and their gait appeared unaffected. Two injections of pH 4.0 saline into one gastrocnemius muscle 2 days apart resulted in a bilateral decrease in the MWT (Fig. 1). All rats completed the study and no complications were observed.

### Experiment 1

An intraperitoneal injection of 300 mg/kg of ROE resulted in a significant increase in the MWT at 15, 30, 45, and 60 min bilaterally, compared with control group (*p* = 0.001, <0.001, 0.001, and 0.017 at ipsilateral site, Fig. 1a, Additional file 1: Table S1; *p* = 0.001, <0.001, 0.001 and 0.019 at contralateral site; Fig. 1b, Additional file 2: Table S2). Compared with the control group, the 100 mg/kg ROE group showed a significant increase in the MWT at 15, 30 and 45 min bilaterally (*p* = 0.013, 0.008 and 0.030 at ipsilateral site, Fig. 1a, Additional file 1: Table S1; *p* = 0.010, 0.017, and 0.042 at contralateral site; Fig. 1b, Additional file 2: Table S2).

**Fig. 2:**
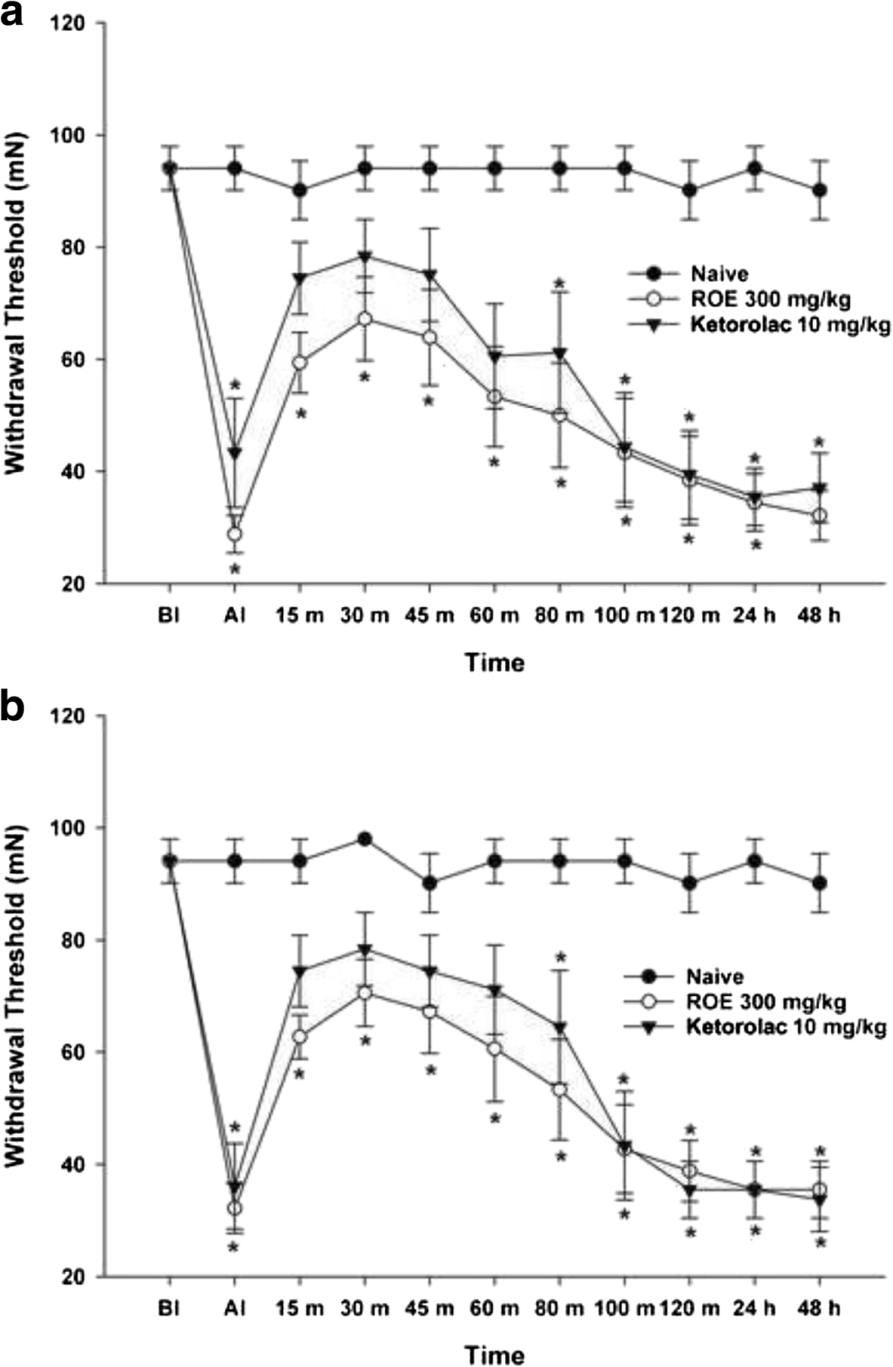
Antinociceptive effect of *Rubus occidentalis* extract 300 mg/kg compared with Naive and Ketorolac 10 mg/kg groups (**a**. ipsilateral site, **b**. contralateral site). ROE, *Rubus occidentalis* extract; BI, before first injection; AI, after second injection, *p < 0.05 compared with the Naive group. Data were analyzed using repeated measures of ANOVA followed by Tukey test

The MWT significantly reduced in Ketorolac 10 mg/kg group at AI, 80, 100, and 120 min, 24 and 48 h bilaterally compared with Naive group (*p* <0.001, 0.029, <0.001, <0.001, <0.001, and <0.001 at ipsilateral site, Fig. 2a; *p* <0.001, 0.035, <0.001, <0.001, <0.001, and <0.001 at contralateral site, Fig. 2b). An intraperitoneal injection of 300 mg/kg of ROE resulted in a significant decrease in the MWT at AI, 15, 30, 45, 60, 80, 100, and 120 min, 24, and 48 h bilaterally compared with Naive gorup (*p* <0.001, 0.002, 0.013, 0.017, 0.003, 0.003, <0.001, <0.001, <0.001, and <0.001 at ipsilateral site, Fig. 2a; *p* <0.001, 0.002, 0.016, 0.012, 0.010, 0.007, <0.001, <0.001, <0.001, and <0.001 at contralateral site, Fig. 2b). There was no significant difference between Ketorolac 10 mg/kg and ROE 300 mg/kg groups bilaterally (Fig. 2).

### Experiment 2

**Fig. 3:**
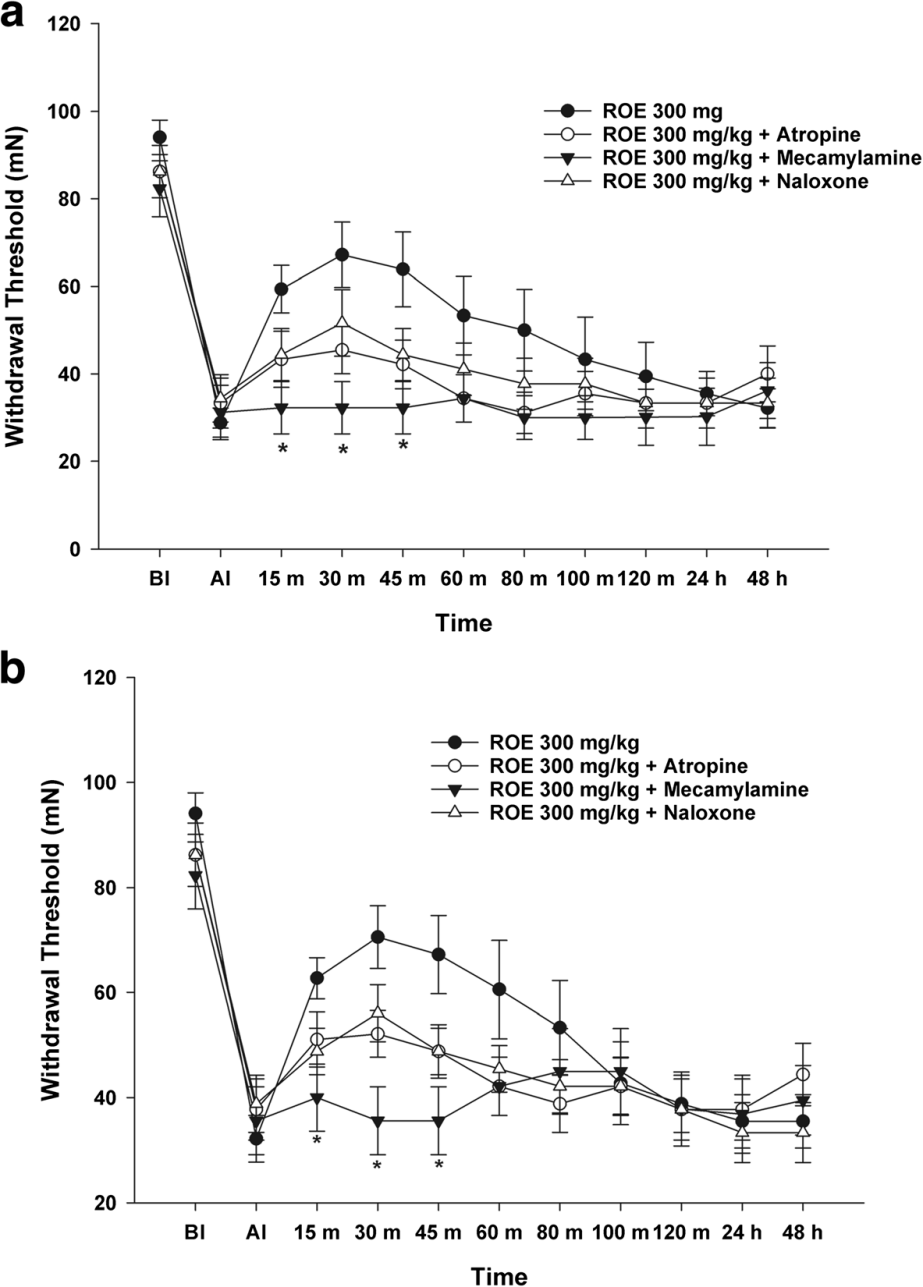
Antinociceptive mechanism of *Rubus occidentalis* extract together with atropine, mecamylamine, or naloxone (**a**. ipsilateral site, **b**. contralateral site). ROE, *Rubus occidentalis* extract; BI, before first injection; AI, after second injection. *p < 0.05 compared with the ROE 300 mg/kg group. Data were analyzed using repeated measures of ANOVA followed by Tukey test

**Fig. 4:**
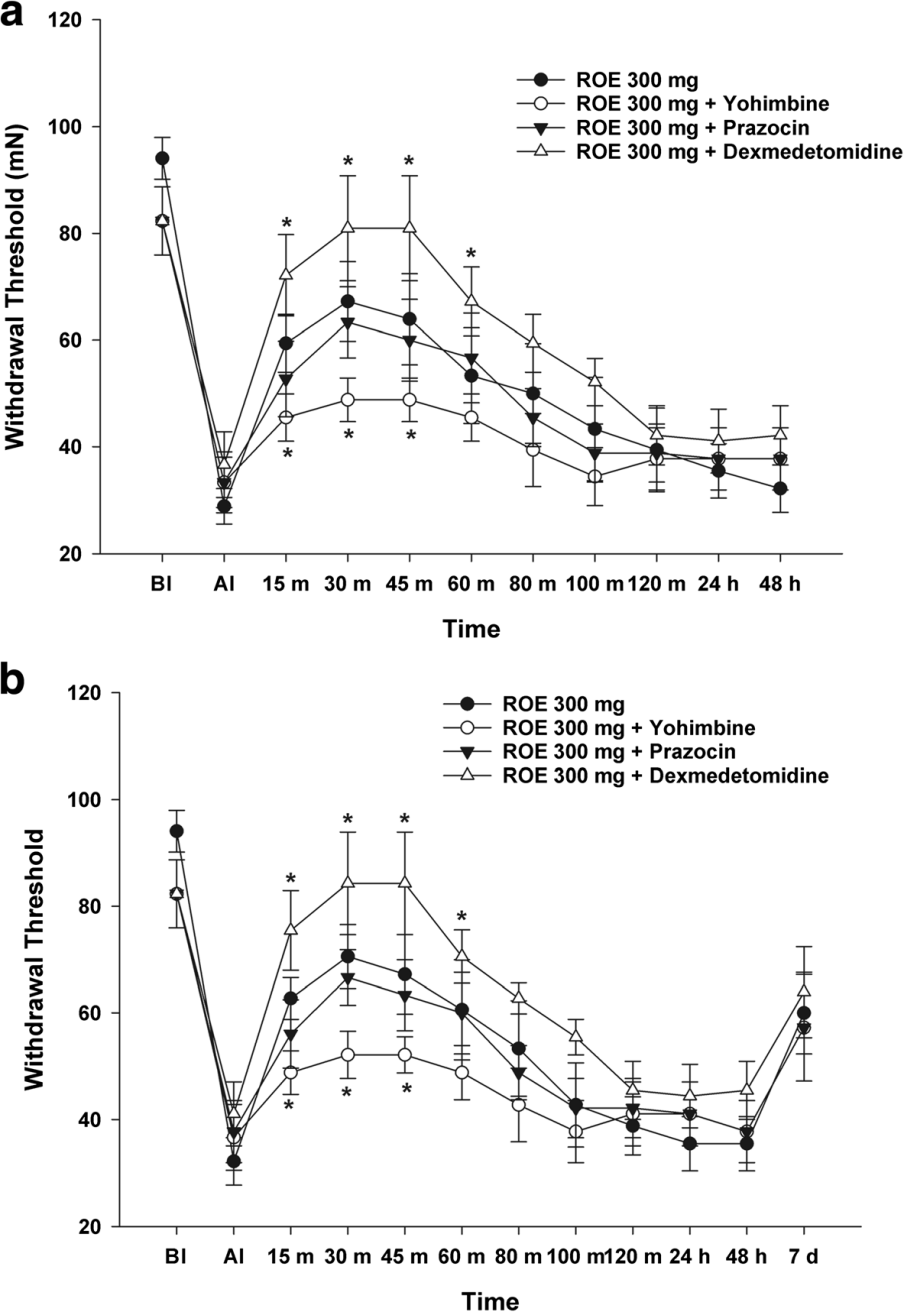
Antinociceptive mechanism of *Rubus occidentalis* extract together with yohimbine, prazosin, or dexmedetomidine (**a**. ipsilateral site, **b**. contralateral site). ROE, *Rubus occidentalis* extract; BI, before first injection; AI, after second injection. *p < 0.05 compared with the ROE 300 mg/kg group. Data were analyzed using repeated measures of ANOVA followed by Tukey test

In the ROE with mecamylamine group, the MWT significantly decreased bilaterally at 15, 30, and 45 min compared with the ROE only group (*p* = 0.014, 0.004 and 0.009 at ipsilateral site, Fig. 3a; *p* = 0.020, 0.001 and 0.004 at contralateral site, Fig. 3b). The MWT decreased bilaterally in the ROE with yohimbine group at 15, 30 and 45 min compared with the ROE only group (*p* = 0.018, 0.014 and 0.035 at ipsilateral site, Fig. 4a; *p* = 0.021, 0.007 and 0.044 at contralateral site, Fig. 4b), while it increased bilaterally in the ROE with dexmedetomidine group at 15, 30, 45 and 60 min (*p* = 0.009, 0.001 0.001 and 0.038 at ipsilateral site, Fig. 4a; *p* = 0.035, 0.012, 0.011 and 0.041 at contralateral site, Fig. 4b).

### Rota-Rod test of motor function

The administration of ROE showed no significant effect on motor performance as measured by the Rota-Rod test 30 min after intraperitoneal injection of ROE compared with the control group (*p* = 0.815). The times to falling during the Rota-Rod test were 116.67 ± 30.55, 113.00 ± 16.09, 98.00 ± 29.86, 115.33 ± 18.56, 101.00 ± 22.65, 117.33 ± 19.55, and 107.33 ± 17.56 s in the control and 10, 30, 100, 300 mg/kg ROE, Ketorolac 10 mg/kg, and Naive groups, respectively.

## Discussion

In the present study, ROE administrated intraperitoneally was found to have antinociceptive activity against hyperalgesia induced by repeated intramuscular injections of acidic saline, and the strength of the antinociceptive effect was proportional to the dosage of ROE. Furthermore, the antinociceptive effect of ROE was significantly reduced by mecamylamine and yohimbine, and potentiated by dexmedetomidine, suggesting that the nicotinic cholinergic and α2-adrenergic receptors are linked to the analgesic effect of ROE.

Scientists have elucidated the active components in *Rubus* fruits and found that they contain antioxidants such as anthocyanins, phenolic acid, and ellagitannin [3, 21]. *Rubus occidentalis* is particularly rich in anthocyanins [2], which are phenolic compounds that are responsible for the red/purple/black pigmentation of fruits and vegetables and possess potent antioxidant activity [22]. This property is associated with potential benefits for obesity, coronary heart disease, degenerative disorders, and various types of cancer [3, 6]. Since oxidative stress can influence muscle sensitivity, there may be a reciprocal interaction between oxidative stress and skeletal muscle susceptibility to fatigue and pain [8]. Furthermore, free radicals are known to be associated with pain induction in chronic pain conditions by causing a decrease in the threshold of nociceptors locally and thus leading to hyperalgesia [23, 24]. Hence, the antinociceptive effect of ROE for chronic musculoskeletal pain and the high anthocyanin content of *R. occidentalis* might be linked. Previous studies have reported antinociceptive effects of other *Rubus* spp., strengthening our hypothesis [9, 10]. Our study using a rat model of chronic muscle pain showed an antinociceptive effect of ROE, suggesting that ROE might have clinical applications for chronic musculoskeletal pain. Besides, the analgesic effect of ROE was compatible with that of Ketorolac as a reference analgesic drug in present study. Ketorolac is a non-steroidal anti-inflammatory drug that has been available to human use for over 20 years [25]. It has been established as a popular parenteral analgesic in clinical practice. Also, several experimental studies in rats evaluated its analgesic efficacy [26–28]. Thus, similar analgesic effect between ROE and Ketorolac would support the possibility of clinical use of ROE.

Chronic musculoskeletal pain poses great therapeutic challenges for clinicians and imposes high healthcare costs on a national scale, while reducing working time and productivity [1]. Additionally, chronic pain can cause compounding effects by contributing to considerable psychological problems such as depression and anxiety [29]. A number of pharmacologic and non-pharmacologic treatment strategies have been proposed for chronic musculoskeletal pain. However, there is no clearly effective treatment thus far, and the pharmacological agents currently used, such as opioids, non-steroidal anti-inflammatory drugs, anticonvulsants, and antidepressants, have limited effectiveness and safety [1, 30]. It is therefore important to identify new strategies for the treatment of chronic pain. In this respect, our finding that ROE showed an antinociceptive effect on chronic musculoskeletal pain in a rat model could be worthy of notice.

Although the specific mechanisms of chronic musculoskeletal pain are not well understood, the participation of α-adrenergic and cholinergic systems in several painful processes is well established [31–33]. To determine the roles of the α-adrenergic and cholinergic receptors in the antinociceptive effect of ROE against chronic musculoskeletal pain in rats, we pretreated the rats with yohimbine, dexmedetomidine, or prazosin to modulate the α-adrenergic system or mecamylamine or atropine to modulate the cholinergic system. Numerous studies have shown a key role for α2-adrenergic receptor and its agonists in acute and chronic pain [32], and α2-adrenoceptor agonist is strongly linked to an antinociceptive effect. Presynaptic activation of α2-adrenoceptor decreases the release of transmitters from primary afferent fibers that inhibit the transmission of pain information [34]. This is correlated with our finding that the antinociceptive effect of ROE was reversed by yohimbine and increased by dexmedetomidine, which suggests that α2-adrenergic receptor mediates the antinociceptive effect of ROE. Although an analgesic effect can also be induced by α1-adrenoceptor agonist [33], there seemed to be no significant relationship between the analgesic effect of ROE and prazosin in the present study.

Several studies have demonstrated an antinociceptive effect of cholinergic receptor activation. Rowley et al. reported the antinociceptive actions of a nicotinic cholinergic agonist [31], which is associated with our finding that the nicotinic antagonist mecamylamine reversed the antinociceptive effect of ROE. Although muscarinic agonist can also induce an analgesic effect [35], there was no significant effect of atropine in the present study.

There were some limitations of the present study. First, we used a non-inflammatory pain model, but several studies have reported an anti-inflammatory effect of ROE [36, 37]. Given that anti-inflammatory effects can contribute to antinociceptive activity [38], further study using an inflammatory model of chronic pain would provide further information regarding the anti-inflammatory effect of ROE and its therapeutic value for pain management. Besides, ROE can attenuate inflammatory responses by reducing the secretion of proinflammatory cytokines, which can induce pain [39]. However, the development of an animal model mimicking chronic pain is difficult because several factors can contribute to chronic pain, unlike acute pain models in which the cause is clear [40]. The acidic saline induced pain model used in the current study has been proposed to have validity as a model of chronic widespread pain conditions in humans [41]. Second, we used ripe *R. occidentalis* fruit in our experiment. Several studies have investigated the fruits of *Rubus* spp. according to their degree of maturity [2, 42]. Further study of the antinociceptive activity of ROEs prepared using *R. occidentalis* fruit at varying stages of maturity would be helpful in the future.

Our study had the strength, notwithstanding these limitations, of being the first experimental study on the effect of ROE on chronic musculoskeletal pain model in rats using a rigorous study protocol.

## Conclusions

*R. occidentalis* showed antinociceptive activity against chronic muscle-induced pain, which may be mediated by α2-adrenergic and nicotinic cholinergic receptors.

## Abbreviations

AI, after second injection; ANOVA, analysis of variance; BI, before first injection; MWT, mechanical withdrawal threshold; ROE, *Rubus occidentalis* extract; rpm, rotation per minute; spp, species

## Additional files

Additional file 1: Table S1. Mechanical withdrawal threshold at ipsilateral site. Rats were injected intraperitoneally with ROE at the indicated doses after the development of hyperalgesia. ROE, *Rubus occidentalis* extract; BI, before injection; AI, after injection. (XLSX 15 kb) Additional file 2: Table S2. Mechanical withdrawal threshold at contralateral site. Rats were injected intraperitoneally with ROE at the indicated doses after the development of hyperalgesia. ROE, *Rubus occidentalis* extract; BI, before injection; AI, after injection. (XLSX 15 kb)

## References

[1] Uhl RL, Roberts TT, Papaliodis DN, Mulligan MT, Dubin AH (2014). Management of chronic musculoskeletal pain. J Am Acad Orthop Surg.

[2] Johnson JL, Bomser JA, Scheerens JC, Giusti MM (2011). Effect of black raspberry (Rubus occidentalis L.) extract variation conditioned by cultivar, production site, and fruit maturity stage on colon cancer cell proliferation. J Agric Food Chem.

[3] Kaume L, Howard LR, Devareddy L (2012). The blackberry fruit: a review on its composition and chemistry, metabolism and bioavailability, and health benefits. J Agric Food Chem.

[4] Dai J, Patel JD, Mumper RJ (2007). Characterization of blackberry extract and its antiproliferative and anti-inflammatory properties. J Med Food.

[5] Krauze-Baranowska M, Majdan M, Halasa R, Glod D, Kula M, Fecka I, Orzel A (2014). The antimicrobial activity of fruits from some cultivar varieties of Rubus idaeus and Rubus occidentalis. Food Funct.

[6] Wang SY, Lin HS (2000). Antioxidant activity in fruits and leaves of blackberry, raspberry, and strawberry varies with cultivar and developmental stage. J Agric Food Chem.

[7] Tian Q, Giusti MM, Stoner GD, Schwartz SJ (2006). Urinary excretion of black raspberry (Rubus occidentalis) anthocyanins and their metabolites. J Agric Food Chem.

[8] Vecchiet J, Cipollone F, Falasca K, Mezzetti A, Pizzigallo E, Bucciarelli T, De Laurentis S, Affaitati G, De Cesare D, Giamberardino MA (2003). Relationship between musculoskeletal symptoms and blood markers of oxidative stress in patients with chronic fatigue syndrome. Neurosci Lett.

[9] Choi J, Lee KT, Ha J, Yun SY, Ko CD, Jung HJ, Park HJ (2003). Antinociceptive and antiinflammatory effects of Niga-ichigoside F1 and 23-hydroxytormentic acid obtained from Rubus coreanus. Biol Pharm Bull.

[10] Niero R, Cechinel Filho V, Souza MM, Montanari JL, Yunes RA, Delle Monache F (1999). Antinociceptive activity of niga-ichigoside F1 from Rubus imperialis. J Nat Prod.

[11] Sluka KA, Kalra A, Moore SA (2001). Unilateral intramuscular injections of acidic saline produce a bilateral, long-lasting hyperalgesia. Muscle Nerve.

[12] Yokoyama T, Maeda Y, Audette KM, Sluka KA (2007). Pregabalin reduces muscle and cutaneous hyperalgesia in two models of chronic muscle pain in rats. J Pain.

[13] Kilkenny C, Browne WJ, Cuthill IC, Emerson M, Altman DG (2010). Improving bioscience research reporting: the ARRIVE guidelines for reporting animal research. PLoS Biol.

[14] Jung KA, Han D, Kwon EK, Lee CH, Kim YE (2007). Antifatigue effect of Rubus coreanus Miquel extract in mice. J Med Food.

[15] Lee S, You Y, Yoon HG, Kim K, Park J, Kim S, Ho JN, Lee J, Shim S, Jun W (2011). Fatigue-alleviating effect on mice of an ethanolic extract from Rubus coreanus. Biosci Biotechnol Biochem.

[16] Gainok J, Daniels R, Golembiowski D, Kindred P, Post L, Strickland R, Garrett N (2011). Investigation of the anti-inflammatory, antinociceptive effect of ellagic acid as measured by digital paw pressure via the Randall-Selitto meter in male Sprague–Dawley rats. Aana j.

[17] Barocelli E, Calcina F, Chiavarini M, Impicciatore M, Bruni R, Bianchi A, Ballabeni V (2004). Antinociceptive and gastroprotective effects of inhaled and orally administered Lavandula hybrida Reverchon “Grosso” essential oil. Life Sci.

[18] Viana AF, Maciel IS, Motta EM, Leal PC, Pianowski L, Campos MM, Calixto JB (2011). Antinociceptive Activity of Trichilia catigua Hydroalcoholic Extract: New Evidence on Its Dopaminergic Effects. Evid Based Complement Alternat Med.

[19] Leite Gde O, Fernandes CN, de Menezes IR, da Costa JG, Campos AR (2012). Attenuation of visceral nociception by alpha-bisabolol in mice: investigation of mechanisms. Org Med Chem Lett.

[20] Shou-Shi W, Ting-Ting S, Ji-Shun N, Hai-Chen C (2015). Preclinical efficacy of Dexmedetomidine on spinal cord injury provoked oxidative renal damage. Ren Fail.

[21] Skrovankova S, Sumczynski D, Mlcek J, Jurikova T, Sochor J (2015). Bioactive Compounds and Antioxidant Activity in Different Types of Berries. Int J Mol Sci.

[22] Kong JM, Chia LS, Goh NK, Chia TF, Brouillard R (2003). Analysis and biological activities of anthocyanins. Phytochemistry.

[23] Khalil Z, Liu T, Helme RD (1999). Free radicals contribute to the reduction in peripheral vascular responses and the maintenance of thermal hyperalgesia in rats with chronic constriction injury. Pain.

[24] Fulle S, Mecocci P, Fano G, Vecchiet I, Vecchini A, Racciotti D, Cherubini A, Pizzigallo E, Vecchiet L, Senin U (2000). Specific oxidative alterations in vastus lateralis muscle of patients with the diagnosis of chronic fatigue syndrome. Free Radic Biol Med.

[25] Boyer KC, McDonald P, Zoetis T (2010). A novel formulation of ketorolac tromethamine for intranasal administration: preclinical safety evaluation. Int J Toxicol.

[26] Malmberg AB, Yaksh TL (1992). Antinociceptive actions of spinal nonsteroidal anti-inflammatory agents on the formalin test in the rat. J Pharmacol Exp Ther.

[27] Chellman GJ, Lollini LO, Dorr AE, DePass LR (1994). Comparison of ketorolac tromethamine with other injectable nonsteroidal anti-inflammatory drugs for pain-on-injection and muscle damage in the rat. Hum Exp Toxicol.

[28] Zhang Y, Shaffer A, Portanova J, Seibert K, Isakson PC (1997). Inhibition of cyclooxygenase-2 rapidly reverses inflammatory hyperalgesia and prostaglandin E2 production. J Pharmacol Exp Ther.

[29] Crofford LJ (2015). Psychological aspects of chronic musculoskeletal pain. Best Pract Res Clin Rheumatol.

[30] Kroenke K, Krebs EE, Bair MJ (2009). Pharmacotherapy of chronic pain: a synthesis of recommendations from systematic reviews. Gen Hosp Psychiatry.

[31] Rowley TJ, Payappilly J, Lu J, Flood P (2008). The antinociceptive response to nicotinic agonists in a mouse model of postoperative pain. Anesth Analg.

[32] Crassous PA, Denis C, Paris H, Senard JM (2007). Interest of alpha2-adrenergic agonists and antagonists in clinical practice: background, facts and perspectives. Curr Top Med Chem.

[33] Tasker RA, Connell BJ, Yole MJ (1992). Systemic injections of alpha-1 adrenergic agonists produce antinociception in the formalin test. Pain.

[34] Lavand’homme PM, Eisenach JC (2003). Perioperative administration of the alpha2-adrenoceptor agonist clonidine at the site of nerve injury reduces the development of mechanical hypersensitivity and modulates local cytokine expression. Pain.

[35] Duttaroy A, Gomeza J, Gan JW, Siddiqui N, Basile AS, Harman WD, Smith PL, Felder CC, Levey AI, Wess J (2002). Evaluation of muscarinic agonist-induced analgesia in muscarinic acetylcholine receptor knockout mice. Mol Pharmacol.

[36] Montrose DC, Horelik NA, Madigan JP, Stoner GD, Wang LS, Bruno RS, Park HJ, Giardina C, Rosenberg DW (2011). Anti-inflammatory effects of freeze-dried black raspberry powder in ulcerative colitis. Carcinogenesis.

[37] Medda R, Lyros O, Schmidt JL, Jovanovic N, Nie L, Link BJ, Otterson MF, Stoner GD, Shaker R, Rafiee P (2015). Anti inflammatory and anti angiogenic effect of black raspberry extract on human esophageal and intestinal microvascular endothelial cells. Microvasc Res.

[38] Sommer C, Kress M (2004). Recent findings on how proinflammatory cytokines cause pain: peripheral mechanisms in inflammatory and neuropathic hyperalgesia. Neurosci Lett.

[39] Liu H, Talalay P (2013). Relevance of anti-inflammatory and antioxidant activities of exemestane and synergism with sulforaphane for disease prevention. Proc Natl Acad Sci U S A.

[40] DeSantana JM, da Cruz KM, Sluka KA (2013). Animal models of fibromyalgia. Arthritis Res Ther.

[41] Green PG, Alvarez P, Gear RW, Mendoza D, Levine JD (2011). Further validation of a model of fibromyalgia syndrome in the rat. J Pain.

[42] Lee JE, Cho SM, Park E, Lee SM, Kim Y, Auh JH, Choi HK, Lim S, Lee SC, Kim JH (2014). Anti-inflammatory effects of Rubus coreanus Miquel through inhibition of NF-kappaB and MAP Kinase. Nutr Res Pract.

